# Disruption of mycorrhizal extraradical mycelium and changes in leaf water status and soil aggregate stability in rootbox-grown trifoliate orange

**DOI:** 10.3389/fmicb.2015.00203

**Published:** 2015-03-18

**Authors:** Ying-Ning Zou, A. K. Srivastava, Qiu-Dan Ni, Qiang-Sheng Wu

**Affiliations:** ^1^Institute of Root Biology/College of Horticulture and Gardening, Yangtze UniversityJingzhou, China; ^2^National Research Centre for CitrusMaharashtra, India

**Keywords:** aggregation, citrus, mean weight diameter, mycorrhizas, water potential

## Abstract

Arbuscular mycorrhizas possess well developed extraradical mycelium (ERM) network that enlarge the surrounding soil for better acquisition of water and nutrients, besides soil aggregation. Distinction in ERM functioning was studied under a rootbox system, which consisted of root+hyphae and root-free hyphae compartments separated by 37-μm nylon mesh with an air gap. Trifoliate orange (*Poncirus trifoliata*) seedlings were inoculated with *Funneliformis mosseae* in root+hyphae compartment, and the ERM network was established between the two compartments. The ERM network of air gap was disrupted before 8 h of the harvest (one time disruption) or multiple disruptions during seedlings acclimation. Our results showed that mycorrhizal inoculation induced a significant increase in growth (plant height, stem diameter, and leaf, stem, and root biomass) and physiological characters (leaf relative water content, leaf water potential, and transpiration rate), irrespective of ERM status. Easily-extractable glomalin-related soil protein (EE-GRSP) and total GRSP (T-GRSP) concentration and mean weight diameter (MWD, an indicator of soil aggregate stability) were significantly higher in mycorrhizosphere of root+hyphae and root-free hyphae compartments than non-mycorrhizosphere. One time disruption of ERM network did not influence plant growth and soil properties but only notably decreased leaf water. Periodical disruption of ERM network at weekly interval markedly inhibited the mycorrhizal roles on plant growth, leaf water, GRSP production, and MWD in root+hyphae and hyphae chambers. EE-GRSP was the most responsive GRSP fraction to changes in leaf water and MWD under root+hyphae and hyphae conditions. It suggests that effect of peridical disruption of ERM network was more impactful than one-time disruption of ERM network with regard to leaf water, plant growth, and aggregate stability responses, thereby, implying ERM network aided in developing the host plant metabolically more active.

## Introduction

Plant rhizosphere often inhabits a kind of beneficial soil fungi from the phylum Glomeromycota known as arbuscular mycorrhizal fungi (AMF), which can colonize the roots of ~80% land's plants to form arbuscular mycorrhizas (AMs) (Smith and Read, 2008). Extraradical mycelium (ERM) network in the soil, a key component of AMs, extends far beyond the root zone to acquire labile forms of soil mineral nutrients and water for the host plant (Selosse et al., 2006) and many infochemicals from one plant to another (Simard and Durall, 2004; Achatz and Rillig, 2014). ERM has been reported to facilitate the free exchange of nutrients and water within the fungal mycelium (Barto et al., 2012). However, no such evidence showing the role of ERM on water absorption is available.

AMs have been observed to affect water movement into the host plant, by regulating the plant hydration and other related physiological processes (Miransari, 2010). Earlier, Wu et al. (2013) proposed that water transport of ERM is possibly more important with soils exposed to water deficit than saturated soil conditions. Allen (2007) in a study demonstrated that extraradical hyphae of AMs are hydrophilic in nature and impart an additional advantage to absorb water from the soil, eventually to deliver into the host plant through hyphal tips, joining through root apoplastic pathway (Smith and Smith, 2011). Water influxes of the ERM remained active even during drought stress condition (Egerton-Warburton et al., 2008). The ERM contribution of water transfer to host plant is only a small percentage (Egerton-Warburton et al., 2007). Khalvati et al. (2005) found that as low as 4% of water in hyphal compartment was transferred to the root compartment by ERM.

Besides absorption of water, ERM can release a glycoproteinaceous substance, glomalin, into the rhizosphere (Wright and Upadhyaya, 1998). As proposed by Wright and Upadhyaya (1998), soil glomalin was extracted by citrate buffer for 0.5 or 1 h at 121°C and autoclaving and quantified by the Braford assay. However, the high-temperature extraction did not exclude all heated-stable proteins and thus lead to the co-extraction of proteins of both AMF as well as non-AMF origin (Purin and Rillig, 2007). As a result, Rillig (2004) proposed the term popularly known as glomalin-related soil protein (GRSP) to replace glomalin in soils. GRSP represents the heat stability and insoluble nature in its native state (Steinberg and Rillig, 2003). In addition, GRSP has been reported to prevent loss of water from the soil exposed to various abiotic stresses (Nichols, 2008; Zou et al., 2014), thereby, regulating the water relations within soil–plant continum. GRSP also promotes soil aggregation through a glue function to bind together macroaggregates (>0.25 mm size) of different sizes, especially in coarse textured soils (Spohn and Giani, 2010). However, in field, the ERM network is often disrupted by soil tillage (Curaqueo et al., 2011). It is not clear whether such disruption of ERM network makes any effects on soil aggregate stability and plant water status.

Trifoliate orange [*Poncirus trifoliata* (L.) Raf.] is a widely used rootstock of citrus plants in orchard all over the world. This rootstock shows less root hair and thus strongly depends on AMs. In this background, the present study was undertaken to determine: (i) the functionings of ERM on plant water status and soil aggregate stability in root+hyphae and root-free hyphae zones of AMF-inoculated trifoliate orange on the basis of a rootbox system, and (ii) evaluate if the short-time or continuous disruption of ERM network affect the soil–plant relation.

## Materials and methods

### Experimental setup

**Figure 1:**
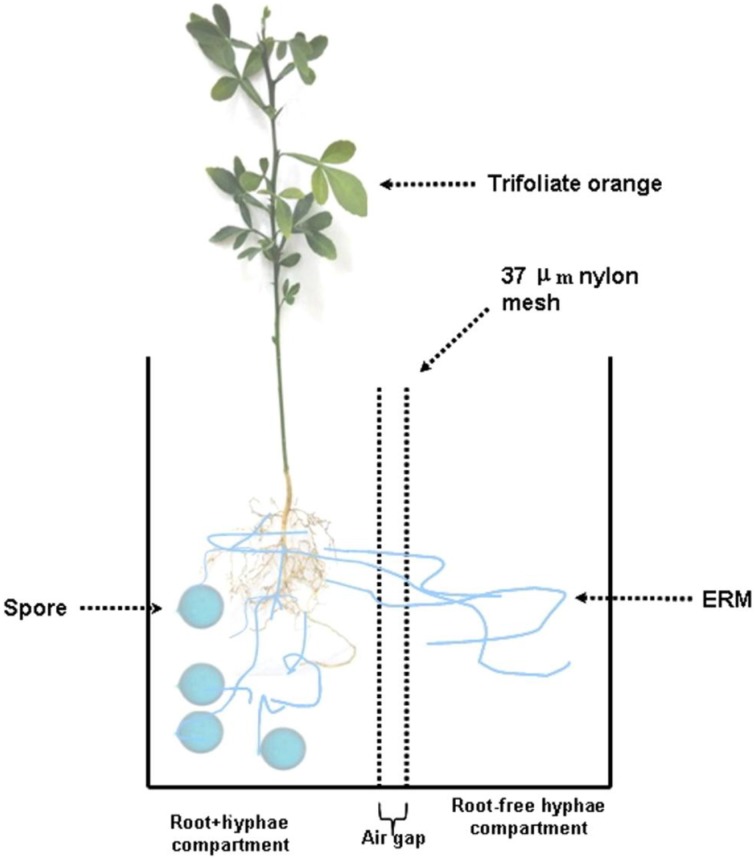
**Schematic diagram of a two-compartment rootbox system to grow trifoliate orange seedlings**. The rootbox was divided into root+hyphae compartment and root-free hyphae compartment through 37–μm nylon mesh, which has the ability to allow mycorrhizal hyphae, but not roots, to enter the compartment. An air gap (1 cm width) was created using two layers of nylon mesh to avoid additional diffusion between root+hyphae and hyphae compartments. Two trifoliate orange seedlings were planted in the root+hyphae compartment, where *Funneliformis mosseae* was inoculated.

Seeds of trifoliate orange were surface sterilized with 70% of ethanol for 10 min and germinated in autoclaved sands at 28°C. Five-leaf-old seedlings with uniform size were used as the plant material. A rootbox system (20 × 10 × 18 cm, length × width × height) made of polyvinyl chloride was used to form an ERM network. The rootbox system has been schematically shown (Figure 1). Simply, the rootbox system was divided into two equal compartments through 37–μm nylon mesh. The mesh had the ability to allow mycorrhizal hyphae, but not roots, to enter the compartment. So, the rootbox was divided into root+hyphae compartment and root-free hyphae compartment. An air gap (1 cm width) was created using two layers of nylon mesh to avoid additional diffusion between root+hyphae and hyphae compartments. Two trifoliate orange seedlings were transplanted into a root+hyphae compartment.

Both root+hyphae and root-free hyphae compartments were supplied with 1.4 kg of the autoclaved (121°C, 0.11 Mpa, 2 h) soil (Xanthi-Udic Ferralsol, FAO system) collected from citrus orchard of the Yangtze University campus (30°36′N, 112°14′E), where the 25-year-old citrus plant (*Citrus unshiu* cv. Guoqing 1) grafted on trifoliate orange was planted. The soil of root+hyphae compartment was mixed with or without *Funneliformis mosseae* (Nicol. and Gerd.) Schüß ler and Walker [BGC XZ02A] (~1000 spores) prior to transplanting. While, non-AMF treatment was also supplied with the same amount of autoclaved (121°C, 0.11 Mpa, 2 h) inoculum plus 2 mL inoculum filtrate (25 μm filter) to keep similar microbial communities except the AM fungus. The spores of this strain were purchased from the Bank of Glomeromycota in China and propagated with white clover for 16 weeks in sand culture.

The seedlings were grown in a glass greenhouse with a photon flux density of 982 μmol/m^2^/s, 27/20°C (day/night) and a relative humidity of 80% during April 1 to September 1, 2013. To ensure the normal growth of the seedlings, 50 mL standard Hoagland solution was supplied at weekly interval into each compartment.

### Experimental design

The experiment consisted of four treatments viz., (i) non-AMF, the plants of the root compartment carrying the inoculation without AMF; (ii) ERM, the plants of the root compartment carrying inoculation with AMF; (iii) ERM-O, plants of the root compartment carrying inoculation with AMF, coupled with an ERM network of air gap cut only one time before 8 h of the harvest; and (iv) ERM-W, the plants of the root compartment carrying inoculation with AMF, coupled with an ERM network of air gap cut at weekly interval after 14 days of AMF inoculation. Meanwhile, a 18-cm-length knife was used to disrupt the 2–5 μm diameter ERM (Smith and Smith, 2011) of air gap between the two compartments. Each treatment was replicated three times in a completely randomized block arrangement. The seedlings were harvested after 4 months of the acclimation.

### Variables determination

At harvest, seedlings were divided into shoots and roots, whose biomass was determined at 75°C for 48 h. The subsample (1-cm long) of fresh roots from four treated seedlings was cleared by 10% KOH solution at 95°C for 1.5 h and stained with 0.05% trypan blue in lactophenol at room temperature for 5 min (Phillips and Hayman, 1970). Root mycorrhizas were observed in microscope, and root AM colonization was calculated as the percentage of AM colonized root length against total observed root length. The nylon meshes collected were cut into 2 × 2 cm size, stained with 0.05% trypan blue in lactoglycerol for 3 min, and observed in microscope.

Relative water content (RWC) of the fourth fully expanded top leaf was estimated with the following formula (Bajji et al., 2001): RWC (%) = (FW–DW)/(SW–DW) × 100, where FW stands for fresh weight, DW for dry weigth at 75°C for 48 h, and SW for saturated weight after leaf rehydration for 24 h. Leaf water potential (leaf *Ψ*) was recorded before plant harvest using a PSΨ PRO Water Potential System with a leaf hygrometer (L-51A-SF, WESCOR).

Transpiration rate (*Tr*) of the fifth fully expanded top leaf was determined by the Li-6400 Portable Photosynthesis System (Li-Cor, Lincoln, USA) on a sunny day between 9:00 and 10:00 am, based on 400 μmol/mol [CO_2_].

Two GRSP fractions, easily-extractable glomalin-related-soil-protein (EE-GRSP) and difficultly-extractable glomalin-related-soil-protein (DE-GRSP), were extracted by the protocol suggested by Wu et al. (2014). EE-GRSP was extracted with 8 mL 20 mM citrate (pH 7.0) and 1.0 g air-dried soil at 121°C and 0.11 Mpa for 30 min and centrifuged at 10,000 g for 3 min. While, DE-GRSP was extracted with both 8 mL 50 mM citrate (pH 8.0) and the remaining residues of EE-GRSP extraction at 121°C and 0.11 Mpa for 60 min and centrifuged at 10,000 g for 3 min. The protein concentrations of these supernatants were analyzed by the Bradford assay with bovine serum albumin as the standard. Total glomalin-related soil protein (T-GRSP) was the sum of EE-GRSP and DE-GRSP.

In order to analyze water-stable aggregate (WSA), the air-dried soil samples were shaken through a series of sieves at 2.00, 1.00, 0.50, and 0.25 mm size based on the wet-sieving method (Kemper and Rosenau, 1986). The WSA fraction was expressed as a percentage of WSA size against total dry soil sample. The aggregate stability was characterized by mean weight diameter (MWD), worked out using the following formula as described by Kemper and Rosenau (1986):

$MWD=\sum_{i\;=\;1}^{n}XiWi$, where, *Xi* is the diameter of the *i* sieve opening (mm), *Wi* is the proportioni of the *i* size fraction in the total sample mass, and (*n* = 4) is the number of size fractions.

### Statistical analysis

Data were analyzed statistically for variance (ANOVA) using SAS software (SAS Institute Inc., Cary, NC, USA). The Duncan's multiple range test was performed to compare significant differences among treatments at *P* < 0.05.

## Results and discussion

### Mycorrhizal colonization and ERM

**Figure 2:**
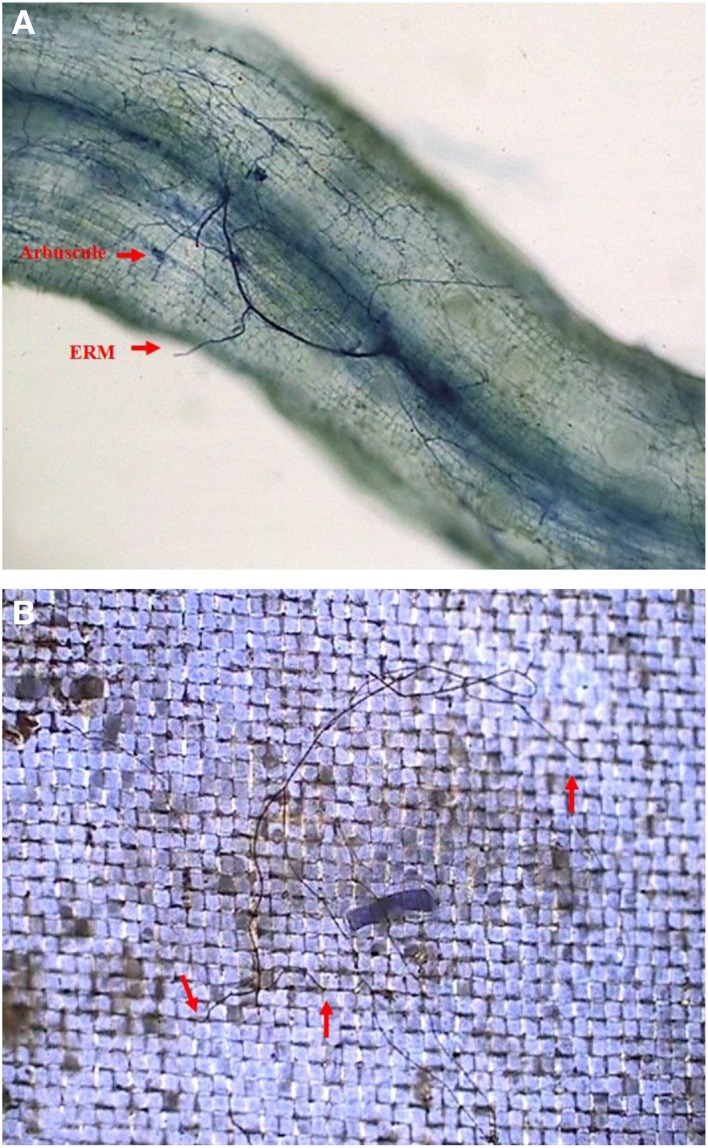
**Root mycorrhizal colonization (A) and extraradical mycelium (B, red arrow shows the entry of extraradical mycelium into 37 μm nylon-mesh) in trifoliate orange (*Poncirus trifoliata*) seedlings inoculated with *Funneliformis mosseae* and grown in 37 μm nylon-mesh separated root/hyphae compartments**.

In root+hyphal compartment, root mycorrhizal colonization of the seedlings varied from 32.1 to 49.5% (Figure 2A; Table 1). ERM was found in the 37–μm of nylon mesh, and rest of the ERM passed through the nylon mesh (Figure 2B), confirming the formation of an ERM network between two distinctly different compartments. Root mycorrhizal colonization was not significantly different when compared between ERM-O and ERM treatments. On the other hand, mycorrhizal colonization significantly decreased by 35.2% with broken ERM as compared with intact ERM treatment (Table 1). These observations implied that the destruction of ERM network adversely affected the magnitude of root colonization. Previous studies showed higher AMF activity under less intensive treatment, such as non-tillage (corresponding ERM treatment only) compared to conventional tillage (corresponding to disruption of ERM) (Curaqueo et al., 2010). Mycorrhizal mycelium have an ability to supply nutrients and energy to infecting hyphae, where nutrients are stored within distal hyphal growing points (Evans and Miller, 1990). This could lead to an AMF inoculum less effective, thus, strongly decreasing the root AM colonization.

**Table 1:** **Root mycorrhizal colonization and plant growth performance of trifoliate orange inoculated with or without *Funneliformis mosseae* and grown in 37 μm nylon-mesh separated root/hyphae compartments**.

**Treatment**	**Root AM colonization (%)**	**Stem diameter (cm)**	**Plant height (cm)**	**Biomass (g FW/plant)**		
				**Leaf**	**Stem**	**Root**
Non-AMF	0.0±0.0c	2.51±0.09b	25.2±1.4b	0.16±0.01c	0.74±0.10c	0.69±0.04c
ERM	49.5±1.9a	3.08±0.10a	33.6±1.8a	0.24±0.01a	1.36±0.03a	0.91±0.07a
ERM-O	47.8±1.5a	2.97±0.11a	32.6±2.2a	0.24±0.01a	1.30±0.05a	0.89±0.00a
ERM-W	32.1±2.2b	3.13±0.13a	31.9±0.8a	0.21±0.01b	0.91±0.09b	0.80±0.04b

*Data (means ± SD, n = 3) followed by different letters between treatments indicate significant differences at 5% level. Abbreviation: ERM, the plants of the root compartment carrying inoculation with Funneliformis mosseae; ERM-O, the plants of the root compartment carrying inoculation with F. mosseae, coupled with an ERM network of air gap cut only one time before 8 h of the harvest; ERM-W, the plants of the root compartment carrying inoculation with F. mosseae, coupled with an ERM network of air gap cut at weekly interval after 14 days of inoculation; Non-AMF, the plants of the root compartment carrying the inoculation without F. mosseae*.

### Growth performance

Citrus is a highly AMF dependent crop (Srivastava et al., 2002). Our study indicated that compared with non-AMF control, AMF inoculation significantly increased all the growth characteristics viz., plant height, stem diameter, and leaf, stem, and root biomass production of the host plant, irrespective of ERM status (Table 1). The beneficial effect of AMF on growth performance of the host plant has been largely attributed to improvement in the mobilization and uptake of nutrients such as P, Fe, Mn, Zn, etc., particularly in poor soils (Beltrano et al., 2013). Our study also observed a non-significant difference in plant growth performance between the ERM and the ERM-O treatment, suggesting that the destruction of ERM once before the harvest of seedlings imposed only a slight change in mycorrhizal functioning. On the other hand, mycorrhizal seedlings recorded significantly lower leaf, stem, and root biomass production under the ERM-W condition than under the ERM or ERM-O condition. Severe destruction of the ERM as executed through the ERM-W treatment was directly responsible for lower efficiency of AMF via lower root AM colonization.

### Leaf water status

**Figure 3:**
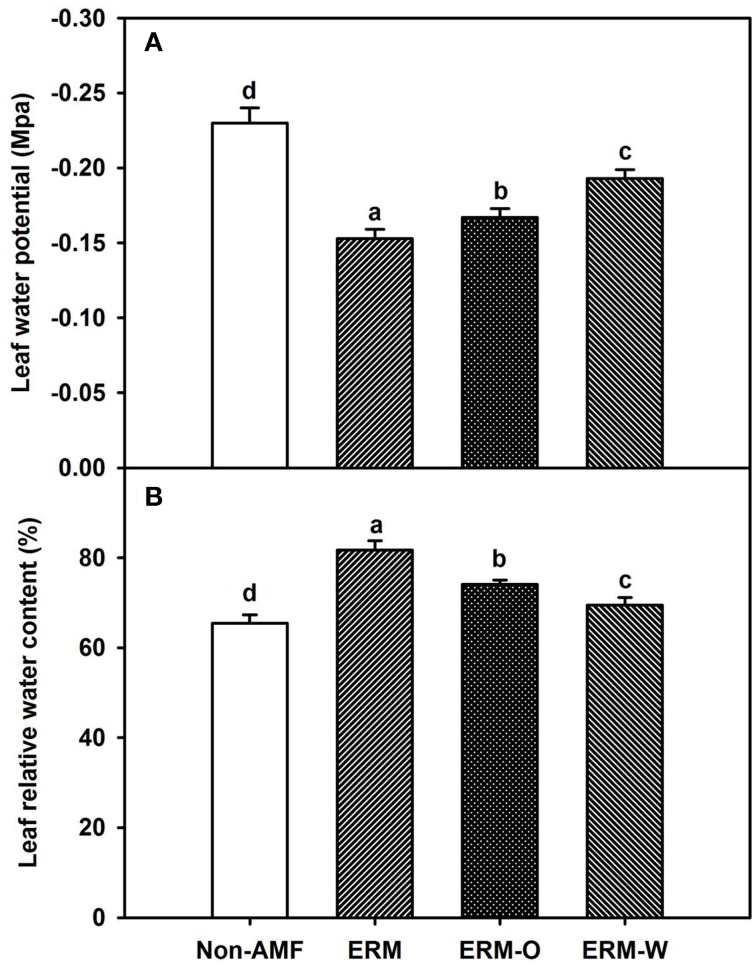
**Effects of extraradical mycelium (ERM) status on leaf water potential (A) and leaf relative water content (B) in trifoliate orange (*Poncirus trifoliata*) seedlings inoculated with or without *Funneliformis mosseae* and grown in 37 μm nylon-mesh separated root/hyphae compartments**. Data (means ± SD, *n* = 3) followed by different letters above the bars among treatments indicate significant differences at the 5% level. Abbreviation: ERM, the plants of the root compartment carrying inoculation with *Funneliformis mosseae*; ERM-O, the plants of the root compartment carrying inoculation with *F. mosseae*, coupled with an ERM network of air gap cut only one time before 8 h of the harvest; ERM-W, the plants of the root compartment carrying inoculation with *F. mosseae*, coupled with an ERM network of air gap cut at weekly interval after 14 days of inoculation; Non-AMF, the plants of the root compartment carrying the inoculation without *F. mosseae*.

**Figure 4:**
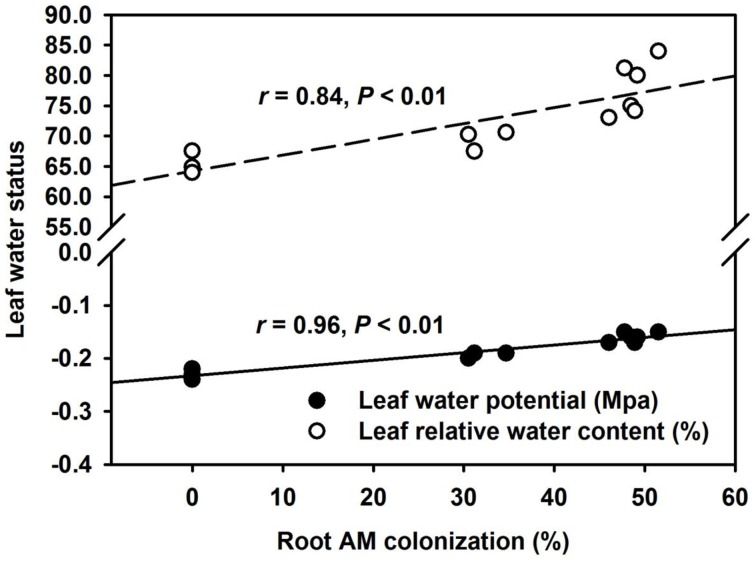
**Linear regression between root AM colonization and leaf water potential or leaf relative water content in trifoliate orange (*Poncirus trifoliata*) seedlings inoculated with or without *Funneliformis mosseae* and grown in 37 μm nylon-mesh separated root/hyphae compartments (*n* = 12)**.

AMF inoculation has been widely reported to enhance tolerance to abiotic stresses including drought stress (Huang et al., 2014; Zou et al., 2014). Our study showed that all the AMF treatments, significantly increased leaf Ψ (Figure 3A) and leaf RWC (Figure 3B) than non-AMF treatments. Better leaf water status in the AMF seedlings was ascribed to an elevation in the functioning of ERM on water uptake, since the hydrophilic nature of mycorrhizal hyphae facilitated absorption of water from the soil and consequently delivery into the host plant (Allen, 2007). In the light of distinctly smaller (2–5 μm) diameter of AM hyphae than root diameter (10–20 μm), the former has easy access through small soil pores (that predominantly retain water), to explore soils's reserve water zone (Smith and Smith, 2011). Correlation studies further showed a significantly (*P* < 0.01) positive correlation of root AM colonization with leaf Ψ and RWC (Figure 4). The ERM-O treatment showed 6.3 and 9.2% significantly lower RWC and leaf Ψ than the ERM treatment, respectively (Figure 3). Likewise, RWC and leaf Ψ under the ERM-W treatment were 11.6 and 26.1% significantly lower than under the ERM treatment and 5.7 and 15.6% significantly lower than under the ERM-O treatment. Allen (2006) estimated that the rate of water transport from ERM to the root was 100 nL H_2_O/h/hyphal infection point. It seems that the rate of hyphal water transport is drastically reduced upon disruption of ERM, thus, resulting in the decrease in leaf water status in the host plant. Further studies in this regard will need to clarify the issue involved in absorption of water by ERM through tracking fluorescent dyes (Egerton-Warburton et al., 2008).

### Leaf transpiration rate (*Tr*)

**Figure 5:**
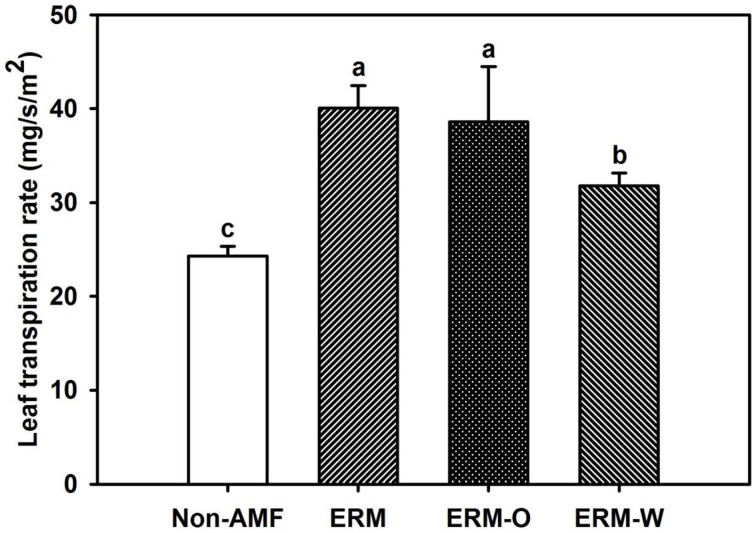
**Effect of extraradical mycelium (ERM) status on leaf transpiration rate in trifoliate orange (*Poncirus trifoliata*) seedlings inoculated with or without *Funneliformis mosseae* and grown in 37 μm nylon-mesh separated root/hyphae compartments**. Data (means ± SD, *n* = 3) followed by different letters above the bars among treatments indicate significant differences at the 5% level. Abbreviation: ERM, the plants of the root compartment carrying inoculation with *Funneliformis mosseae*; ERM-O, the plants of the root compartment carrying inoculation with *F. mosseae*, coupled with an ERM network of air gap cut only one time before 8 h of the harvest; ERM-W, the plants of the root compartment carrying inoculation with *F. mosseae*, coupled with an ERM network of air gap cut at weekly interval after 14 days of inoculation; Non-AMF, the plants of the root compartment carrying the inoculation without *F. mosseae*.

Leaf water status was closely related with leaf *Tr*, since the latter mediates the balance between transpiration stream and water uptake by roots (Rapparini and Penuelas, 2014). In the present study, leaf *Tr* in AMF seedlings was significantly higher than non-AMF seedlings (Figure 5), which would induce a higher hydraulic lift from hyphae in mycorrhizal treatment (Egerton-Warburton et al., 2008). The ERM-O treatment in our studies showed no change in leaf *Tr* compared to the ERM treatment. Once the ERM was disturbed, leaf *Tr* of the ERM-W treated seedlings was 20.6% significantly lower than the ERM treated seedlings, suggesting that continuous disruption of ERM network could decrease leaf *Tr*, subsequently inducing the decrease of hydraulic lift from ERM, finally resulting in lower leaf water status in the ERM-W treated seedlings.

### Changes of GRSP fractions

GRSP acts as a gluing material to stabilize soil aggregates into varying dimensions (Wright and Upadhyaya, 1998). In the present study, the rhizosphere soil of all the AMF seedlings recorded significantly higher GRSP fractions viz., EE-GRSP, DE-GRSP, and T-GRSP within the root+hyphae compartment (Table 2), suggesting that synthesis of GRSP fractions was induced by AMF inoculation. In addition, DE-GRSP concentration was significantly higher under the ERM-W treatment than either the ERM or the ERM-O treatment. It is claimed that DE-GRSP is originated from turnover of EE-GRSP, but the turnover time is not clearly known (Wu et al., 2014). In the root+hyphae compartment, continuous destruction of ERM might be another triggering factor stimulating the turnover of EE-GRSP into DE-GRSP, besides strong contribution of deciduous ERM in accelerating the transformation of EE-GRSP into DE-GRSP.

**Table 2:** **Concentrations of glomalin-related soil protein (GRSP, mg/g DW) fractions in root+hyphae and hyphae compartment of trifoliate orange inoculated with or without *Funneliformis mosseae* and grown in 37 μm nylon-mesh separated root/hyphae compartments**.

**Treatment**	**Root+hyphae compartment**			**Root-free hyphae compartment**		
	**EE-GRSP**	**DE-GRSP**	**T-GRSP**	**EE-GRSP**	**DE-GRSP**	**T-GRSP**
Non-AMF	0.494 ± 0.006b	0.536 ± 0.004c	1.030 ± 0.003c	0.470 ± 0.011c	0.564 ± 0.005a	1.035 ± 0.013c
ERM	0.584 ± 0.007a	0.563 ± 0.002b	1.147 ± 0.009b	0.522 ± 0.014b	0.572 ± 0.017a	1.094 ± 0.030b
ERM-O	0.581 ± 0.010a	0.556 ± 0.017b	1.137 ± 0.027b	0.515 ± 0.008b	0.564 ± 0.013a	1.079 ± 0.012b
ERM-W	0.592 ± 0.010a	0.594 ± 0.012a	1.186 ± 0.004a	0.586 ± 0.016a	0.582 ± 0.006a	1.168 ± 0.019a

*Data (means ± SD, n = 3) followed by different letters between treatments indicate significant differences at 5% level. Abbreviation: ERM, the plants of the root compartment carrying inoculation with Funneliformis mosseae; ERM-O, the plants of the root compartment carrying inoculation with F. mosseae, coupled with an ERM network of air gap cut only one time before 8 h of the harvest; ERM-W, the plants of the root compartment carrying inoculation with F. mosseae, coupled with an ERM network of air gap cut at weekly interval after 14 days of inoculation; Non-AMF, the plants of the root compartment carrying the inoculation without F. mosseae*.

In the root-free hyphae compartment, we also observed a significantly higher EE-GRSP and T-GRSP concentration in the AMF seedlings as compared to non-AMF seedlings (Table 2). Similarly, the ERM-W treatment induced significantly higher EE-GRSP and T-GRSP concentration than either the ERM-O or ERM treatment. It could be a possibility that the ERM-W treatment resulted in ERM slipping off, producing some new glomalin, namely, EE-GRSP (Koide and Peoples, 2013), thereby, conferring a higher EE-GRSP level in the hyphae compartment. However, no significant difference with respect to DE-GRSP was observed in the mycorrhizosphere of hyphae compartment, irrespective of ERM status. The ERM, ERM-O, and ERM-W treatments represented two contrasting responses of DE-GRSP between root+hyphae and root-free hyphae compartments, primarily on account of the presence or absence of root exudates, which had various traits of several enzymatic activities, possibly involved in the turnover of EE-GRSP into DE-GRSP (Badri and Vivanco, 2009; Muratova et al., 2009).

**Figure 6:**
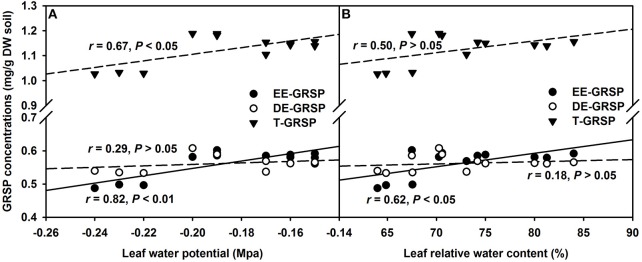
**Linear regression between leaf water potential (A) or leaf relative water content (B) and three glomalin-related soil protein (GRSP) fractions of root+hyphae compartment in trifoliate orange (*Poncirus trifoliata*) seedlings inoculated with or without *Funneliformis mosseae* (*n* = 12)**.

Amongst the three GRSP fractions, EE-GRSP and T-GRSP were significantly positively correlated with leaf Ψ (Figure 6A). While, only EE-GRSP was significantly positively correlated with RWC (Figure 6B). These observations further showed that GRSP acts as protectant in form of coating on fungal hyphae and soil particles to prevent the evaporation loss of water (Nichols, 2008; Zou et al., 2014). Interestingly, only EE-GRSP displayed significant effect on leaf water status compared to DE-GRSP. Possibly, EE-GRSP is a most juvenile form of GRSP released by the AMF hyphae and therefore is more active; while, DE-GRSP is originated from EE-GRSP turnover and more recalcitrant in action (Koide and Peoples, 2013). EE-GRSP, therefore, possesses more impactful behavior in regulating the soil moisture availability to host plants compared to DE-GRSP.

### WSA Distribution and Aggregate Stability

Aggregation of soil particles into different sizes is an important process in soil organic carbon (SOC) stabilization (Andruschkewitsch et al., 2014). Soil aggregation can be influenced by various factors, such as SOC, biota, clay, ionic bridging, and carbonates (Spohn and Giani, 2010). AMF have shown to play significant role in soil aggregate stability through both mycorrhizal hyphae network and GRSPs (Wu et al., 2014). In the present study, we observed that the percentage of WSA at 2.00–4.00, 1.00–2.00, and 0.50–1.00 mm sizes in root+hyphae and root-free hyphae compartments was significantly higher within mycorrhizosphe than non-mycorrhizosphere, except WSA at 0.25–0.50 mm size (Table 3). These modifications in WSAs as a result of mycorrhization are directly related to the nature and properties of mycorrhizal hyphae and extent of the release of GRSP, to stabilize macroaggregates by enmeshing soil particles by mycorrhizal hyphae (Kohler-Milleret et al., 2013) and further binding them together through GRSP (Martin et al., 2012).

**Table 3:** **Distribution of water-stable aggregate (WSA) and mean weight diameter (MWD) in 37 μm nylon-mesh separated root/hyphae compartments of trifoliate orange inoculated with or without *Funneliformis mosseae***.

**Treatment**	**Distribution of WSA (%)**				**MWD (mm)**
	**2.00–4.00 mm**	**1.00–2.00 mm**	**0.50–1.00 mm**	**0.25–0.25 mm**	
**ROOT+HYPHAE COMPARTMENT**					
Non-AMF	5.64±1.04c	6.84±0.21d	13.23±1.40b	37.19±1.62ab	0.51±0.03c
ERM	8.58±0.90b	7.69±0.25c	18.21±0.26a	33.93±0.65b	0.64±0.03b
ERM-O	8.54±0.87b	8.21±0.36b	17.95±0.68a	38.74±2.36a	0.66±0.02ab
ERM-W	10.83±1.08a	8.99±0.26a	18.23±2.13a	30.11±2.32c	0.71±0.04a
**ROOT-FREE HYPHAE COMPARTMENT**					
Non-AMF	2.96±0.22c	4.81±0.63c	11.10±0.16c	10.74±1.72a	0.38±0.01c
ERM	5.37±0.33a	7.42±0.30a	17.05±1.33a	10.50±3.42a	0.53±0.00a
ERM-O	5.91±0.35a	7.74±0.68a	17.23±1.32a	11.01±1.04a	0.56±0.03a
ERM-W	4.00±0.50b	6.12±0.21b	13.76±0.41b	10.77±0.02a	0.45±0.02b

*Data (means ± SD, n = 3) followed by different letters between treatments indicate significant differences at 5% level. Abbreviation: ERM, the plants of the root compartment carrying inoculation with Funneliformis mosseae; ERM-O, the plants of the root compartment carrying inoculation with F. mosseae, coupled with an ERM network of air gap cut only one time before 8 h of the harvest; ERM-W, the plants of the root compartment carrying inoculation with F. mosseae, coupled with an ERM network of air gap cut at weekly interval after 14 days of inoculation; Non-AMF, the plants of the root compartment carrying the inoculation without F. mosseae*.

**Figure 7:**
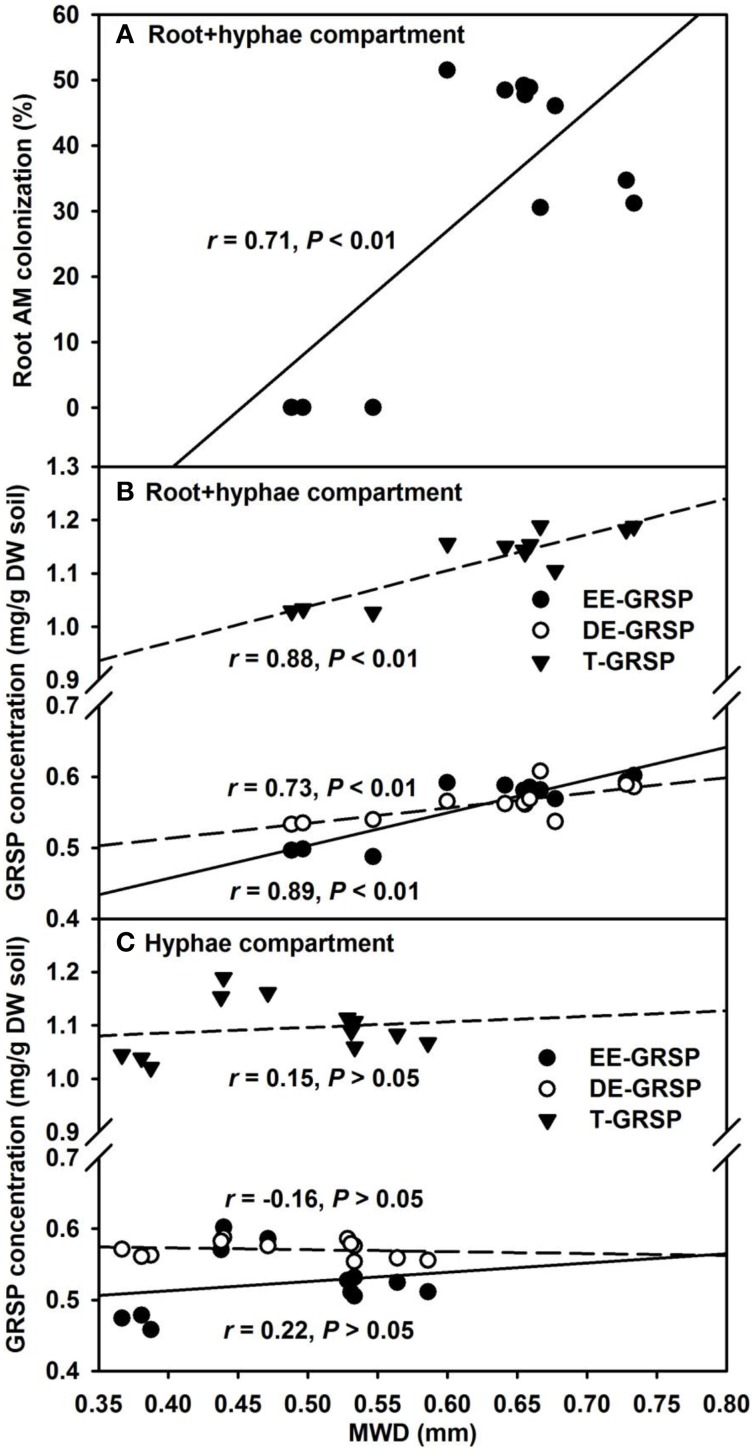
**Linear regression between mean weight diameter (MWD) and root AM colonization **(A)** or three glomalin-related soil protein (GRSP) fractions (**B** and **C**) of 37 μm nylon-mesh separated root/hyphae compartments in trifoliate orange (*Poncirus trifoliata*) seedlings inoculated with or without *Funneliformis mosseae* (*n* = 12)**.

Our results further revealed that mycorrhizal treatments significantly increased MWD under root+hyphae and root-free hyphae conditions, irrespective of ERM status (Table 3). A significantly positive correlation of MWD with root AM colonization, EE-GRSP, DE-GRSP, and T-GRSP (Figures 7A,B) suggested that GRSP fractions and root colonization all together effectively mediated the soil aggregate stability. MWD was strongerly positively correlated with EE-GRSP and T-GRSP than with root colonization and DE-GRSP in root+hyphae compartment. Wu et al. (2014) also reported that in root-free hyphae chamber, EE-GRSP exhibited more strong correlation with MWD than mycorrhizal hyphae. Interestingly, under root-free hyphae conditions, MWD was not significantly correlated with any of the three GRSP fractions (Figure 7C), implying that GRSP is not the main binding agent for soil WSA stability under root-free hyphae conditions (Rillig et al., 2003), because aggregate stability involves integration of different soil physicochemical and biological properties (Borie et al., 2008).

One time disruption of ERM (namely the ERM-O treatment) in our studies failed to alter ERM functioning on aggregate stability (according to MWD value), because no significant difference of MWD was observed between the ERM and the ERM-O treatments (Table 3). However, continuous destruction of ERM (e.g., the ERM-W treatment) altered the functioning, due to which, there was comparatively higher MWD in root+hyphae compartment than in hyphae compartment with the ERM-W vs. the ERM treatment. In root+hyphae compartment, fungal mycelium were in abundance to establish the ERM network for entering hyphae compartment under the ERM-W treatment condition. However, in root-free hyphae compartment, the deciduous mycelium were shortened under the ERM-W, thereby, resulting in functional degradation with respect to binding macroaggregates. Comparing the morphological as well as functional differences of effective mycelium on aggregate stability vs. deciduous mycelium will provide more useful information.

## Conclusion

AMF inoculation displayed highly significant response to plant growth performance, leaf RWC, leaf Ψ, and leaf *Tr* of trifoliate orange seedlings, irrespective of ERM status. Mycorrhizosphere of root+hyphae and root-free hyphae chambers was observed to be richer with respect to concentration of GRSP fractions (except DE-GRSP in hyphae chamber) and MWD over non-mycorrhizosphere. Continuous disruption of ERM network reduced the leaf water status and aggregate stability which adversely affected the growth and diverted the positive response of mycorrhization compared short-time (e.g., 8 h before harvest) disruption of ERM network, with WSA stability remaining unaffected. EE-GRSP as one of major fractions of GRSP contributed most actively in regulating leaf water status as well as WSA stability under root+hyphae and root+free hyphae environment.

## Author contributions

YNZ and QDN were involved in acquisition and analysis of data in the work; QSW and YNZ were involved in experimental design of the work; YNZ and QSW drafted the work; AKS revised it for important intellectual contents. All authors approved the final version.

### Conflict of interest statement

The authors declare that the research was conducted in the absence of any commercial or financial relationships that could be construed as a potential conflict of interest.
